# Supplementary material for: In Vivo Anticoagulant and Antithrombic Activity of Depolymerized Glycosaminoglycan from Apostichopus japonicus and Dynamic Effect–Exposure Relationship in Rat Plasma
Source: Mar Drugs. 2022 Oct 2;20(10):631. doi: 10.3390/md20100631 (PMC9605468; doi:10.3390/md20100631)
Supplement: Supplementary file 1 [file marinedrugs-20-00631-s001.zip › marinedrugs-1928934-supplementary.pdf]

Supplementary Information

In Vivo Anticoagulant and Antithrombic Activity of Depolymerized Glycosaminoglycan from *Apostichopus japonicus* and Dynamic Effect–Exposure Relationship in Rat Plasma

Han Wang [1], Dandan He [1], Linlin Duan [1], Lv Lv [1], Qun Gao [1], Yuanhong Wang [1,2], Shuang Yang [1,2,*] and Zhihua Lv [1,2,*]

[1] Key Laboratory of Marine Drugs, Ministry of Education of China, School of Medicine and Pharmacy, Ocean University of China, Qingdao 266003, China
[2] Laboratory of Marine Drugs and Bioproducts, Pilot National Laboratory for Marine Science and Technology, Qingdao 266237, China
* Correspondence: yangshuang@ouc.edu.cn (S.Y.); lvzhihua@ouc.edu.cn (Z.L.)

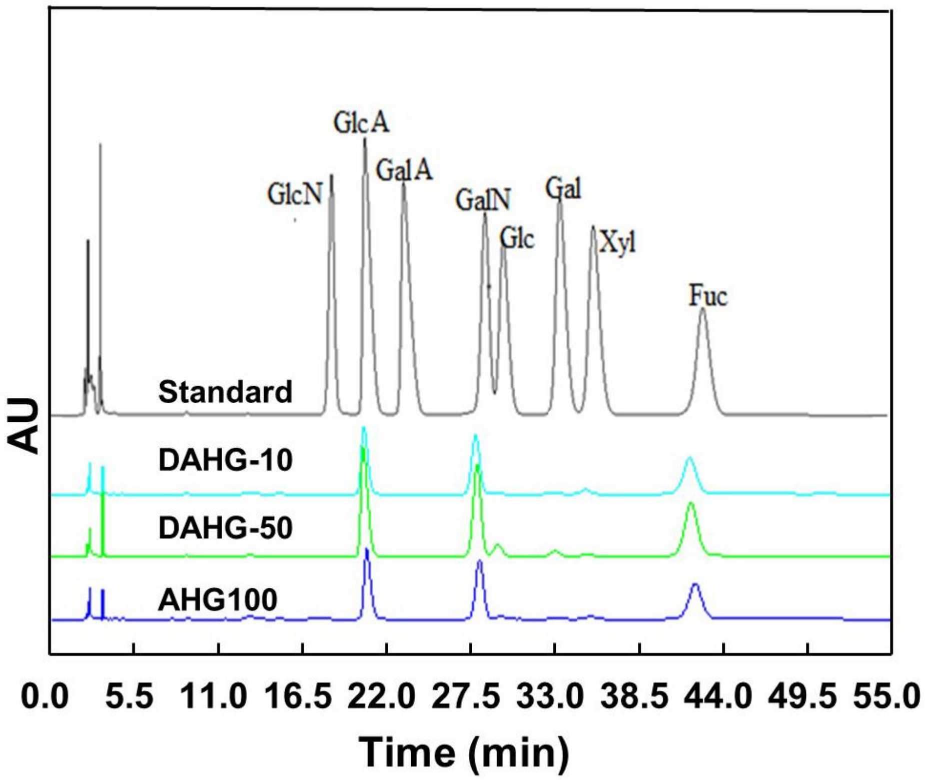

Figure S1. HPLC chromatography of monosaccharide composition analysis.

Note: GlcN (Nacetyl-β-D-Glucosamine); GlcA (β-D-glucuronic acid); GalA (β-D-galacturonic acid); GalN (Nacetyl-β-D-galactosamine); Glc (D-(+)-Glucose); Gal (D-(+)-Galactose); Xyl (D-(+)-Xylose); Fuc (L -(-)-Fucose).

Table S1. The PK-PD modeling parameters of residual FIIa and FXa activity fitted with E_{max} model for DAHG10 in rats.

Parameter	Oral administration (250 mg/kg)		Intravenous injection (5 mg/kg)	
	Value (FIIa)	Value (FXa)	Value (FIIa)	Value (FXa)
E_{max}(%)	97.96	105.59	101.59	104.72
EC_{50} (μg/mL)	57.59	50.75	49.98	59.40
$R_{obs\text{-}pre}$	0.99	0.99	0.99	0.99
AIC	2.56	6.22	27.73	28.65
SC	2.45	6.11	27.89	28.81

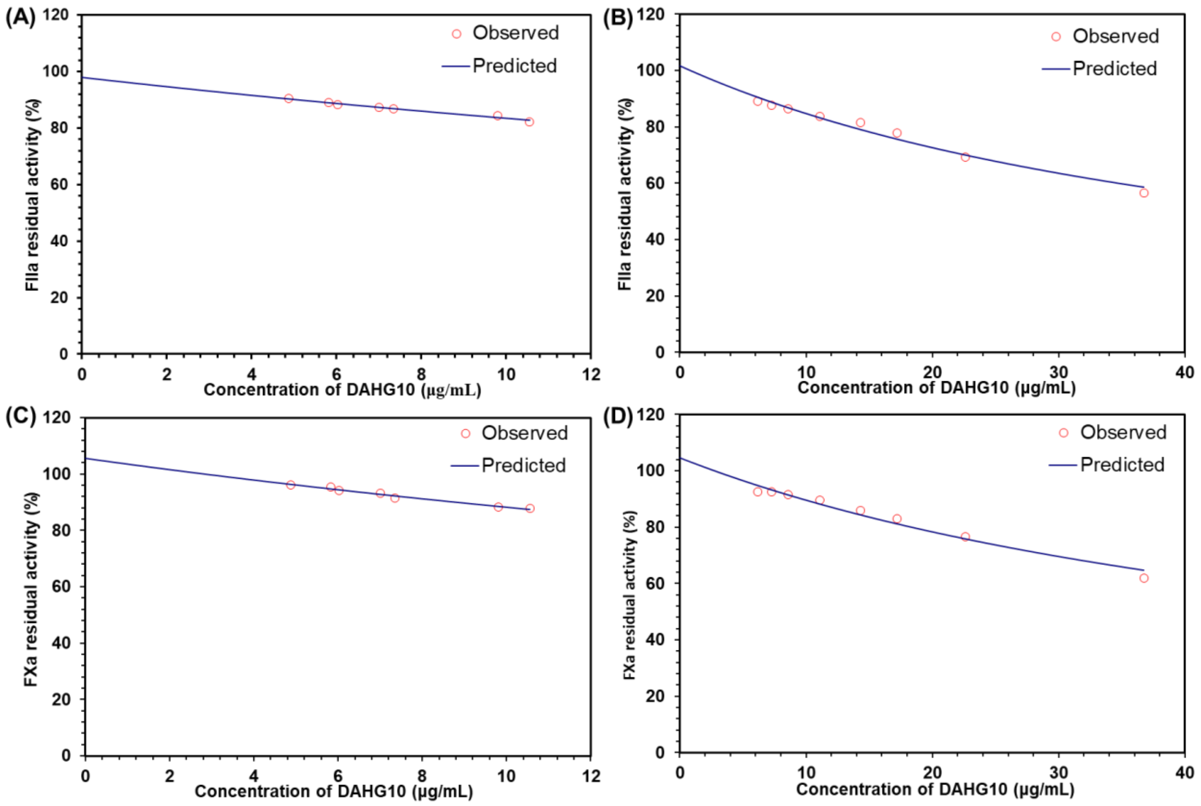

Figure S2. Prediction and observed mean antithrombin effect vs. time profiles for E_{max} (n=4). (A) residual FIIa activity of oral of DAHG10; (B) residual FIIa activity of intravenous injection of DAHG10; (C) residual FXa activity of oral of DAHG10; (D) residual FXa activity of intravenous injection of DAHG10.
